# Sudden cardiac death due to the Wolff–Parkinson–White syndrome: A case report with genetic analysis

**DOI:** 10.1097/MD.0000000000013248

**Published:** 2018-12-21

**Authors:** Mingjie Qiu, Bin Lv, Wei Lin, Jing Ma, Hongmei Dong

**Affiliations:** Department of Forensic Medicine, Tongji Medical College of Huazhong University of Science and Technology, Wuhan, Hubei, PR China.

**Keywords:** arrhythmias, genetic analysis, sudden cardiac death, Wolff–Parkinson–White syndrome

## Abstract

**Rationale:**

The Wolff–Parkinson–White syndrome (WPW) is a benign heart disease with accessory pathways, which can result in cardiac arrhythmias. The purpose of this case report is to introduce a rare case of sudden cardiac death (SCD) with a mild myocardial bridge and a history of WPW.

**Patient concerns:**

A 25-year-old man with known WPW syndrome died at night while sleeping.

**Diagnoses:**

Diagnosis of WPW syndrome is based on typical electrocardiogram findings with a documented dysrhythmia before the victim's death.

**Interventions:**

At autopsy, no traumatic injury or common poisons were found, only a slight myocardial bridge was detected. We performed whole exome sequencing and identified several genetic variations related to SCD.

**Outcomes:**

We considered that the cause of death in this case was SCD in which arrhythmia might play an important role.

**Lessons:**

This case highlights SCD can occur in WPW patients with mild or unrecognized structural abnormality. Postmortem genetic examination can assist the diagnosis of sudden cardiac death, especially when no lethal structural abnormality is found in the decedent.

## 1 Introduction

Wolff–Parkinson–White (WPW) syndrome is a congenital heart conduction disease characterized by the presence of one or more accessory pathways that predispose the patient to frequent episodes of arrhythmias.^[1]^ The occurrence of WPW is rarely associated with other congenital cardiac anomalies.^[2]^ WPW is considered as a benign arrhythmia, but provides a basis for the occurrence of arrhythmias. Patients with WPW syndrome may experience palpitations, dizziness, syncope, congestive heart failure or sudden cardiac death (SCD). In some patients, the first and only manifestation of the disease is SCD.^[2,3]^ The ECG features of WPW include a short PR interval of <0.12 seconds, slurring and slow rise of the initial QRS complex (delta wave), a widened QRS complex with a total duration >0.12 seconds, and an abnormal ventricular repolarization.^[1]^ The ECG finding of pre-excitation is a result of early ventricular depolarization through the accessory pathway. Diagnosis of WPW syndrome is based on typical ECG findings with a documented dysrhythmia. The overall risk of SCD in the WPW syndrome is estimated at 0.1% in asymptomatic patients and 0.3% in symptomatic patients per year.^[4]^ Familial appearance of WPW syndrome displays an autosomal dominant inheritance. The PRKAG2 gene was identified as a possible susceptibility gene in WPW syndrome.^[5]^ PRKAG2 encodes the γ2 subunit of AMP-activated protein kinase (AMPK), which is an important regulator of cardiac metabolism.^[6]^

Herein we reported an autopsy case of sudden death at night while sleeping, with a myocardial bridge in left anterior descending coronary artery and a history of WPW.

## 2 Case report

### 2.1 Case history

A 25-year-old man was charged with robbery and incarcerated in a prison. One night, his cellmates found the man snoring loudly, cyanotic and unresponsive in bed. They called the prison staff and sent him to the infirmary. He presented with pulseless and cardiopulmonary arrest. Cardiopulmonary resuscitation was performed, but he died.

**Figure 1:**
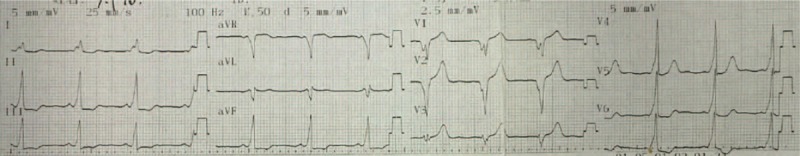
Twelve-lead ECG after conversion to sinus rhythm. Delta waves were observed in aVR, II, V4, V5, and V6. This ECG is indicative of WPW syndrome type B. WPW = Wolff–Parkinson–White syndrome.

His medical records indicated that he had a history of recurrent episodes of palpitation. The symptom occasionally occurred in the past year without obvious cause. It lasted for a few minutes to half an hour and alleviated by itself. The last episode of the symptom was two months ago. The physical examination revealed a normal blood pressure (120/90 mm Hg) and a pulse rate of 160 beats/minute when the symptoms appeared. The 12-lead resting electrocardiogram (ECG) showed paroxysmal tachycardia and pre-excitation syndrome type B (Fig. 1). He was given symptomatic treatment.

### 2.2 Autopsy and histological findings

The man was 172.0 cm in height and 59.0 kg in weight. No significant injuries were observed on external examination except for a few old scars on the knee. The heart weighed 310 g on examination. The left anterior descending artery was located into the myocardial wall for a distance of 1.5 cm and depth was 0.1 cm. The coronary artery revealed no atherosclerotic changes. Focal hemorrhage was observed in the right ventricular myocardium. Other organs did not show any remarkable changes. The toxicology analysis revealed no positive findings.

### 2.3 Genetic analysis

Genomic DNA was isolated from paraffin embedded tissue of the patient and whole exome sequencing was performed to analyze potential genetic variation related to WPW syndrome and SCD. The results showed no exonic mutations in the PRKAG2 gene. Seven potentially pathogenic mutations for SCD, KCNE1, CACNA1C, CASQ2, ANK2, AKAP9, KCNJ5, and TRDN were identified in the patient. The results of the genetic analysis are shown in Table 1.

**Table 1:**
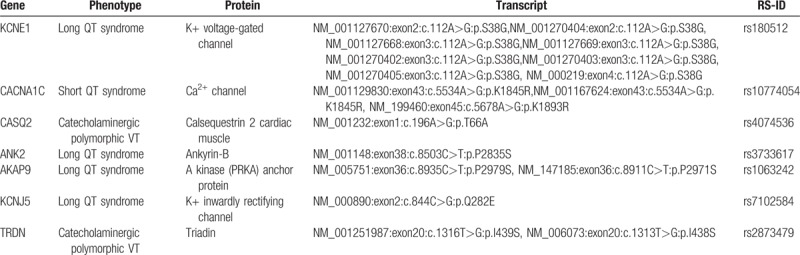
Predisposing gene analysis of SCD by whole exome sequencing.

## 3 Discussion

In the present case, a 25-year-old man died during sleep. No significant abnormality was found in organs except for a myocardial bridge. The frequency of myocardial bridge at autopsy is generally high. It is considered to be pathological when the segment of tunnel is 2 to 3 cm long and 0.2 to 0.3 cm deep.^[7]^ In this case, myocardial bridge was 1.5 cm in length and 0.1 cm in depth without atherosclerotic changes in the intramural part, which was insufficient to explain sudden death. The clinical history of tachycardia, palpitation and ventricular pre-excitation, and ECG finding indicated that he had arrhythmic symptoms accompanied by WPW syndrome. Whole exome sequencing identified mutations in some common SCD-related genes. Thus, it was presumed that the cause of death was SCD, although without lethal structural abnormality. Moreover, WPW with arrhythmias accounted for the death.

The most common arrhythmia in WPW patients is atrioventricular re-entrant tachycardia, which occurs in 80% of cases. Atrial fibrillation is common, occurring in one-third of patients and is a potentially life-threatening arrhythmia. Ventricular fibrillation is the most common cause of sudden death in WPW patients.^[1]^ It is difficult to diagnose arrhythmia related sudden death at postmortem without an antemortem electrocardiogram. For establishing the cause of death, other causes must be excluded, and the history of electrocardiographic abnormalities is essential.

Previous studies have indicated that patients with WPW may suddenly die during sleep.^[8]^ Risk factors for SCD in patients with WPW syndrome are localization of the accessory pathway, emotional or physical stress, additional cardiac structural abnormalities and accompanying symptoms. Sudden death occurred significantly more often in men than in women.^[9]^ A proportion of cases have additional structural abnormalities suggesting that the combination of pre-excitation with additional cardiac pathology may render individuals at higher risk of SCD.^[10]^ In the presented case, a myocardial bridge was found in left anterior descending coronary artery at autopsy. In the presence of the myocardial bridge, myocardial ischemia is more serious when arrhythmias occur. Besides, this concomitant pathology may contribute to atrial fibrillation and result in higher incidence of SCD. Accompanying symptoms such as tiredness, palpitations, syncope or abdominal pains have been considered as alarming signs of sudden death due to WPW syndrome.^[11]^ The patient in our case presented with pre-excitation combined with palpitations and should be on high alert for signs of SCD.

The molecular genetics of WPW has been investigated, but only a single gene PRKAG2 was defined to date.^[6]^ The patient did not carry this mutation. Prior studies have reported that mutations in this gene focused on familial WPW syndrome. Vaughan et al^[12]^ indicated that unlike familial WPW syndrome, mutation of PRKAG2 is not commonly associated with sporadic WPW syndrome. Further studies are needed to identify the genetic basis of common sporadic WPW syndrome. In the presented case, seven mutations in genes associated with SCD were identified in the patient, although the contribution of these mutations to WPW syndrome is unknown. These mutations in ion channel genes lead to abnormalities in ion channel function, which result in abnormal ionic current characteristics via defective channel gating or reduction in sarcolemmal channel expression that eventually leads to arrhythmias.^[13]^

The incidence of sudden death in patients with WPW is extremely low. To the best of our knowledge, this is the only reported case of sudden death in a man with WPW and myocardial bridge. This case highlights that SCD can occur in WPW patients with mild or unrecognized structural abnormality. In such cases, SCD is likely to be caused by arrhythmias although without the terminal ECG. In forensic autopsy cases suspected of SCD caused by arrhythmia, clinical history is needed in addition to detailed autopsy and histopathology examination. Postmortem genetic testing is also helpful in the diagnosis of the specific etiology to explain SCD. In addition, the deceased with WPW syndrome have accessory atrio-ventricular connections in the heart anatomy, but the cardiac autopsy cannot provide a standardized demonstration of accessory pathways at the histological assessment.^[14]^ Hence, the diagnosis of WPW at postmortem relies on typical ECG findings form medical records.

## 4 Ethical standards

The authors declare that all procedures performed in this study were in accordance with the ethical standards of the institutional and/or national research committee and with the 1964 Helsinki declaration and its later amendments or comparable ethical standards. Informed consent was obtained from the parents of the decedent.

## Acknowledgments

The study is funded by the National Nature Science Foundation of China (No. 81471821).

## Author contributions

**Conceptualization:** Mingjie Qiu, Bin Lv, Wei Lin, Hongmei Dong.

**Data curation:** Jing Ma.

**Formal analysis:** Bin Lv.

**Methodology:** Wei Lin.

**Project administration:** Hongmei Dong.

**Software:** Jing Ma.

**Writing – original draft:** Mingjie Qiu, Bin Lv.

**Writing – review & editing:** Hongmei Dong.
